# The Nature and Treatment of Dropsy; Considered Especially in Reference to the Diseases of the Internal Organs of the Body Which Most Commonly Produce It. Parts I. and II. Anasarca and Ascites. With an Appendix, Containing a Translation of the Work of Dr. Geromini on Dropsy, from the Original Italian

**Published:** 1837-04

**Authors:** 


### ART. VIII.

*The Nature and Treatment of Dropsy; considered especially in reference to the Diseases of the internal Organs of the Body which most commonly produce it.* Parts I. and II. *Anasarca and Ascites. With an Appendix, containing a Translation of the Work of* DR. GEROMINI *on Dropsy, from the original Italian.* By EDWARD J. SEYMOUR, M.D., Physician to St. George's Hospital, &c.—*London*, 1837. 8vo. pp. 218.

OPINIONS on the nature of dropsy, like those upon all other frequent and formidable diseases, have continually varied according to the prevailing doctrine of the day. Considerable light was imagined to have been cast on the obscurity of the disease when it was, in one age, pronounced to depend on the cold temperament of the liver, and, in the next, on a supposed contrary condition; or when, in subsequent times, it was explained by a defect in the power of the abdominal viscera of collecting the superfluous humidity; or by chemical, spiritual, and mechanical theories of equal weight, and equally satisfactory to their inventors. It is, then, a matter of no surprise that the peculiar bias of men's minds, in the present age, should have influenced their opinions concerning this disease; nor that, since mere hypothetical reasoning has given way to the study of facts and attempts at philosophical induction, we should have approached more nearly to some real and exact knowledge of the malady. The attention which has been paid to the phenomena of inflammation in the various tissues of the body, since the time of John Hunter, has led to the recognition of the frequent dependence of dropsy on inflammation; and the ardour with which pathological anatomy is now cultivated has induced many to study the connexion between dropsy and organic changes in the various viscera; a connexion recognized faintly by Hippocrates, and very pointedly by the older writers; but only clearly proved and systematically studied in our own days. Exclusive devotion to one system is not the prevailing error at present; and, as far as dropsy is concerned, the general and correct opinion seems to be, that it may depend on very opposite conditions of the whole system, and on very various organic diseases. The title of the work before us seemed to show that Dr. Seymour took this eclectic view of dropsy; and a perusal of it justified the supposition as to the soundness of his general principles.

The first part of the work is devoted to Anasarca; the second, to Ascites.

Dr. Seymour believes dropsy to arise, in most instances, from the return of blood to the right side of the heart being obstructed; and that the secretion of fluid relieves the impaired function of the organ. He illustrates this by the example of œdema of a limb, the simplest form of dropsy, depending on tumours pressing on the veins above the swelling, as in ovarian disease, or in obstruction of the vein itself in phlebitis, as in phlegmasia dolens, &c. In like manner, it is to be expected, when great obstruction exists to the return of blood to the right side of the heart, that the whole venous circulation would be congested, and hence the whole capillary system would secrete fluid as a relief to the obstruction. But, although Dr. Seymour is inclined to the belief that disease of the heart is the most frequent cause of dropsy, he admits its occasional inflammatory origin after eruptive fevers, and the sudden application of cold; and also its dependence on disease of the kidneys.

### *Anasarca from Disease of the Heart.*

1. From Rheumatic Disease of the Heart. The subjects of this disease are generally young, and, with few exceptions, under forty years of age. Dr. S. has seen it in patients of nine years of age. On account of the disease occurring at an early period of life, before excess in the use of spirits, exposure to great changes of temperature, or the advance of years, have injured the soundness of other internal organs, it is the simplest view of the cause and effect. The symptoms are—swelling of the cellular membrane, beginning about the ankles; the face is also swelled, especially the eyelids; an expression of anxiety, even greater than in diseases of the chest in general; pulse generally small and quick, sometimes intermitting, but very small compared with the tumultuous beating of the heart itself, owing to the heart enlarging considerably without a corresponding enlargement of its vessels. At the commencement of the anasarca, there is almost always pain in the region of the heart, often pain in the course of the biceps muscle of the left arm, and cramps in the legs; the patient cannot lie down, and the most easy position is bending over the back of a chair. When the anasarca is very considerable, there is little pain in the chest, as the effusion relieves the heart: the patient then attributes all his sufferings to the dropsy; the legs are tense, face swelled and bluish; great dyspnœa; urine scanty, high coloured; pulse quick and weak; surface cold; short, dry cough, with slight or no expectoration. The pathological appearances are, uniformly, enlargement of the heart itself; the walls, especially those of the left side, thickened, and the cavities enlarged, but not in proportion to the hypertrophy; the pericardium glued together with layers of lymph, rarely recent. In the notes of thirty cases these appearances were uniform, and in all there was well-ascertained evidence of previous rheumatic fever. The prognosis is always unfavorable; but the disease, even when most severe, is more chronic than dropsy from disease of the heart arising from other causes, owing to its occurring in young persons, as the growth of their chest enables the impaired action to be better borne; and also, as it is not the result of excess, spirit drinking, &c., the other viscera are healthy.

*Treatment.* This varies according to the degree of inflammation of the pericardium going on at the time of the anasarca. If it has been immediately preceded by pain in the heart, and some swelling, redness, and pain in the limbs, inflammatory action exists, demanding moderate bleeding in a recumbent position, to prevent fainting, (from which the patient may not recover,) with three grains of calomel and a quarter of a grain of opium every four hours, a blister over the chest, and low diet. The most useful diuretic during this stage is from ten to fifteen grains of nitre, twice daily, in mint water. Dr. Seymour has never found colchicum useful. When the inflammatory symptoms have ceased, and the dropsical symptoms are alone urgent, the great object is to unload the system by diuresis; and here digitalis produces the happiest effect. Dr. Seymour's experience coincides with that of Dr. Withering, who introduced this medicine, and who found it most efficacious in a lax state of the system, when the effusion is great and the action of the heart feeble. The following formula Dr. Seymour has found more certain than any other:

R. Infusi Digitalis, ʒiv.

Liq. Oxymur. Hydrarg. ʒij.

Aq. Menth. Sativ. ʒviij.

Tinct. Cantharidis, m. xxx.

Misce fiat haustus bis terve in die sumendus.

The tincture of cantharides is an active diuretic, and renders almost every other more effectual: it is contra-indicated where disease of the urinary organs is present. The above draught often operates with astonishing rapidity and effect: if, owing to the oxymuriate, it purges, a grain of opium may be given every night. Removing the effusion is obviously merely relieving one symptom; the enlargement of the heart remains, and the patient has a crippled existence. Where poverty obliges him to expose himself again to the causes of the disease, to toil, anxiety, and want, relapses are inevitable.

2. Anasarca from Enlargement of the Heart, principally with increased thickness of the parietes of the left ventricle and the septum between the ventricles.—This form occurs from twenty years of age to sixty, and is almost invariably attributable to intemperance. The anasarca is enormous; the abdominal integuments are so distended as to give the appearance of ascites; occasionally large vesications form on the legs, which burst and discharge serum, affording temporary relief. Pulse generally strong and quick; urine scanty, high coloured, not coagulating by heat or acids; tongue furred. The patient is obliged to be raised in bed, and complains of insupportable oppression. Face swollen, often of a bluish tint; constant thirst; bowels generally natural, and appetite little deranged. Scarifications afford a momentary relief; but, in almost every hospital case, they are succeeded by erysipelas, gangrene, and death. Acupuncture gives less relief, but with somewhat less danger. Those remedies are indicated which, whilst they expel the fluid, will diminish the action of the heart. The first of these is elaterium. This may be given "where the strength is yet unbroken," according to this formula. Bloodletting should be premised.

℞. Elaterii, gr. ½.

Subm. Hydr., Pulv. Capsici, *aa* gr. ij.

Conf. Ros. Canin., q. s. ut ft. pilula; mane sumenda.

This will purge, and often produce severe bilious vomiting, which is very useful. It must be recollected that oppression imitates remarkably true debility. When elaterium has failed, or ceased to produce the due effect, Dr. Seymour has found large doses of cream of tartar, (first recommended by Menghini, an Italian physician,) act very beneficially. He directs an ounce of cream of tartar to be taken every morning in solution; or, if this acts too violently by stool, half an ounce will be sufficient. After the fourth day, its beneficial effects generally commence. The salt requires about thirty parts of boiling water for its solution.

3. Anasarca from increased Size of the Heart, with attenuated parietes. Such cases occur in constitutions broken by intemperance, or debilitated by affliction, long-continued anxiety, great watching, and sometimes after long-continued and profuse evacuations; more frequently in females than in males, and more common in advanced age than the other forms. The pulse is weak, often irregular; face swelled, and ghastly white; the integuments pit on the slightest pressure. The effusion is preceded by dyspnœa, violent palpitations and faintness on quick motion. The dyspnœa is not relieved by the effusion; for not unfrequently the cellular membrane connecting the lobules of the lungs is infiltrated also. Sudden death frequently occurs from rapid effusion into the chest or pericardium. This form is often complicated with dilatation of the arch of the aorta, and atheromatous and bony deposition under the lining membrane. In such cases, besides the symptoms of oppression, there are paroxysms of most severe pain, as in angina pectoris. In this form of the disease digitalis is particularly applicable, which is in accordance with Withering's original observations. It may be given with squill and mercury, thus: Pil. Hydr. gr. iij.; Scillæ exsicc., Pulv. fol. Digitalis, *aa* gr. j.;—or as in the previous formula, with cantharides. This latter medicine Dr. Seymour was led to employ as a diuretic by the recommendation of Sir H. Halford; and its combination with the infusions of digitalis, of spartium, or of pyrola umbellata, he is convinced by repeated experience, produces the most complete diuretic effect. Dr. Seymour makes a digression on the value of cantharides in certain cases of paraplegia in early life: where it has been successful, it has acted as a powerful diuretic, and its utility may be explained by supposing the paralysis to have depended on effusion of serum, gravitating from the brain to the lower part of the spinal canal, which, according to Dr. Baillie, is one cause of the disease. From several years' experience, Dr. Seymour has found the most effectual diuretics stand in the following order of utility:

Infusion of Digitalis, with Tinct. Cantharid.
Nitrate of Potash,
Supertart. of Potash, with Sp. Juniper. c.
Pil. Hydrarg., Pulv. Dig., et Scilla exsicc., in pills.
Acet. et Tinct. Scillæ.
Infus. Pyrolæ umbellatæ.
Infus. Spartii.
Sp. Æth. Nitr.
Sp. Armoraciæ comp.

Dr. Seymour's remaining observations on diuretic medicines are judicious, but do not strike us as containing any thing new. When the anasarca is of recent date, these remedies will often suffice to carry off the water by diuresis, and "careful attention to diet, which should be nutritious but not stimulating, carriage exercise, pure air, and abstinence (as much as possible) from harassing and anxious occupations, will sometimes for years prolong the patient's life; but, where the swellings are of many months' continuance, little is to be expected except relief. Even here, life may be prolonged for a considerable time, under the most unfavorable circumstances, among the opulent. Not so with the poorer class of patients, in whom, in this country, intemperance has been the most active cause of their malady: when relieved of the fluid, which they consider to be the disease, they speedily return to spirit-drinking; and, if there be yet power in the system to secrete a sufficient quantity of fluid, they are again affected by their former swellings." (P.56.)

Sudden subsidence of the effusion, unpreceded by a gradually increased flow of urine, or by some evacuations, is a very fatal symptom. In several cases which fell under Dr. Seymour's observation the patients never survived more than a month after this sudden subsidence of the effusion. Dr. S. attributes the event to the vital powers being no longer sufficiently strong to afford relief to the obstructed circulation by producing secretion from the capillaries.

Such is an analysis of Dr. Seymour's views on Anasarca depending on diseases of the heart. That which strikes us most forcibly is the scanty information on diagnosis. It appears to have been Dr. Seymour's object to shew that various forms of anasarca requiring various modifications in treatment are dependent on different diseases of the heart. As far as it regards the treatment of these different forms of dropsy he is peculiarly minute, and his remarks on the actions of medicines are valuable; but he has somewhat strangely omitted to give sufficient information on a preliminary matter which is certainly of considerable importance, the symptoms by which we are to be guided in the administration of these various remedies. We will begin with rheumatic disease of the heart, by which Dr. S. means chronic pericarditis, which is almost always attended with hypertrophy; in thirty cases in which this occurred, Dr. S. does not mention valvular disease, nor does he allude to it in the whole chapter. The symptoms on which the diagnosis is to be founded are—" pulse generally small and quick, sometimes intermitting, but very small compared with the action going on within the thorax; a tumultuous beating of the whole organ; this smallness of the pulse, compared with the violence in the action of the heart itself, arises from the inordinate growth of the organ, without a corresponding enlargement of the vessels of the heart."

We think it hardly necessary to mention that such symptoms are also common to contraction of one or more of the orifices of the heart, and induration of the valves, and especially so when to these local signs are superadded such a general symptom of impeded circulation as anasarca, and a bluish tint of the complexion. The patients are said by Dr. Seymour to be under forty years of age; but this is of little importance in the diagnosis, as out of forty-four cases of diseased valves reported by Bouillaud there were no less than thirty-three under fifty years of age, and some of these were young children. The previous attack of rheumatism is by no means a decisive diagnostic symptom, as valvular disease is repeatedly traced to the same source. The only chance of distinguishing the two diseases is by the stethoscope; and if there is that importance in making a diagnosis which even Dr. Seymour attaches to diagnosis by separately discussing the treatment of anasarca depending on different diseases of the heart, what excuse can be made for the omission? Again: the only diagnostic mark given of hypertrophy of the heart is "a strong and quick pulse," and of dilatation of the heart "a weak, compressible, and often irregular pulse." Laennec, whose accuracy in these matters few are disposed to question, strongly affirmed that in diseases of the heart the pulse was a most deceptive symptom, and that in the highest degree of hypertrophy it was as common to find the pulse weak as to find it strong. A weak, compressible, and *often* irregular pulse is a partial guide to treatment, but certainly is common to many diseases; even if the pulse alone indicated these diseases in their simplest form, yet all practical men are aware that such characters of the pulse are not to be expected when there is a complication of visceral diseases, which must have been the case with patients whose constitutions (according to Dr. S.) were broken down by excess and misery. The practical importance which Dr. S. attaches to his divisions of anasarca, is that dropsy with hypertrophy is a more active disease, and with dilatation more passive, and consequently the treatment must be modified accordingly. But, as he founds his diagnosis on the pulse merely, which alone is a deceptive symptom, can his statements be regarded or proved, or his practical inferences be estimated as of any peculiar value?

Dr. Seymour adopts the theory of anasarca being generally produced by an impediment to the circulation of the blood through the heart, but his views on the nature of this obstruction do not appear to us to be altogether sound. He merely alludes cursorily to diseases of the valves; he only describes minutely anasarca depending on hypertrophy, and dilatation of the heart; but these alone are not sufficient mechanical obstacles to the circulation to explain in numerous cases the reason of the effusion; they can only be regarded as permanent predisposing causes having great influence, but not as exciting causes. A patient may have for years hypertrophy of the heart with or without adherent pericardium, or he may have dilatation of the ventricles without any dropsical symptoms whatever; but if such a patient should be attacked with bronchitis, or affected with any other complication disturbing the circulation, he may then have anasarca. The disease of the heart was the great predisposing cause, rendering him more liable to bronchial inflammation, but this latter was the exciting cause. We need not apologize for quoting the following passage from M. Bouillaud's work on the heart, reviewed in a former Number, (British and Foreign Review, No. IV. p. 335,) as it bears particularly on one part of this subject. "In uncomplicated hypertrophy of the heart, the respiration is not materially affected till the organ has acquired such a volume as to encroach on the lung; yet the majority of writers on cardiac diseases have ascribed to hypertrophy, or active aneurism, such symptoms as the following: a violet hue of the face, and general congestion of the venous capillaries; passive dropsies and passive hemorrhages, dyspnœa, &c.; but these are, in reality, only so many signs of mechanical obstacle to the circulation, and indicate disease in the orifices, valves, &c."

The next chapter is headed "Cases of Anasarca occurring without organic disease of an internal organ," and these cases are referred to three causes. 1. To cold applied to the body; 2d, to eruptive diseases, especially scarlatina; and 3d, to debility, in chlorotic females, or from hemorrhage. But in his first and second divisions, Dr. Seymour may be accused of begging the question in assuming them to be independent of organic disease. The sudden application of cold or wet is so rarely followed by anasarca, and so commonly productive of suppression of the perspiration, that it may be well doubted whether this latter effect alone satisfactorily explains the effusion; there must be some peculiar predisposition to the affection; and may not this depend on latent organic disease, or, at any rate, an unusual susceptibility of some internal organ to take an organic disease? Dr. Seymour admits that in some of these cases the urine is albuminous; and this alone would lead to the suspicion that occasionally they may depend on renal disease. A case in point has been recently published by Dr. Mateer, of Belfast, in an excellent paper,* which we shall analyze in our Selections from British Journals. The patient, a robust girl of seventeen, became anasarcous over the whole body, after exposure to wet: her urine was albuminous. She was bled locally and generally, saline diuretics were given, the urine became less albuminous, and in six weeks she was able to walk out; but she relapsed without any known cause, and in ten days died. On dissection, her kidnies were found to be in a state of granulation, and all other organs of the body healthy. With regard to anasarca following scarlatina, although Dr. Seymour places it under the head of Anasarca without organic disease, yet he confesses that its pathology is not well understood, and quotes P. Frank in support of the obscurity. But Dr. S. does not allude to the probability of its connexion with disease of the kidneys. The fact of the coagulability of the urine in many of these cases was observed by Dr. Wells and Dr. Blackall, many years ago, and has since been confirmed by other observers: if then in these cases there was anasarca and albuminous urine without renal disease, they would be exceptions to that coincidence which Dr. Bright has pointed out, and which subsequent observation has proved to be almost invariable. We do not doubt that the link which is now wanting will be supplied; we have before alluded to a paper in the Edinburgh Medical and Surgical Journal, in which Mr. Hamilton states that in several fatal cases of dropsy, after scarlatina, he discovered that yellow mottled appearance of the kidney, which now both at home and abroad bears the name of its discoverer, Bright. In the only case published by Dr. Mateer, of scarlatina followed by anasarca and

* Edinburgh Medical and Surgical Journal, Jan. 7, 1837; p. 73.

terminating fatally, the kidneys were alone diseased; and they were soft, livid, "having somewhat the appearance of hepatized lung;" a condition supposed by Dr. Bright to be a stage preceding that of granulation.

The treatment recommended by Dr. Seymour is, to bleed if the patient is strong, and to purge freely, either with calomel, followed by senna and crystals of tartar; or instead of senna, with a teaspoonful of an electuary composed of jalap two drachms, of supertart. potassæ half an ounce, with the same quantity of honey. If the patient is weakly, and his strength broken by previous delicate health and severe attacks of illness, diuretics should be given combined with bitters: as the infusion of digitalis and of gentian with liquor potassæ; or a mixture composed of a pint of the decoction of the pyrola umbellata, and half a drachm of tinct. lyttæ, two ounces of which to be taken every four hours.

3. Anasarca in chlorotic females, or after great loss of blood. Dr. Seymour explains the swelling of the ankles and legs, and the puffy state of the face, on the supposition that the blood stagnates in the right side of the heart, which organ is weak and unable to circulate the blood to the extremities. In thus explaining almost all cases of dropsy according to the theory of mechanical obstacle at the heart, it may be questioned whether Dr. Seymour is not rather one-sided: he somewhat undervalues the powers of the capillary vessels, and their independence of the heart: surely the weak condition of the blood itself and the debility of the capillary vessels would explain the phenomenon of swollen ankles as satisfactorily as the questionable hypothesis of the stagnation of blood in the right side of the heart.

The fifth chapter is headed "Anasarca from disease of the Kidneys," and it contains additional testimony to the coincidence pointed out by Dr. Bright between albuminous urine of low specific gravity and disease of the kidneys: Dr. Seymour has watched this test for many years in the wards of St. George's Hospital, and he has invariably found, "where cases with coagulable urine and low specific gravity proved fatal, that the kidneys presented a fac-simile of the representation given of them in this disease by Dr. Bright, in his Hospital Reports." (P. 73.)

"About half the cases of dropsy admitted into St. George's Hospital, in my experience, have coagulable urine: and in about one-third of such of these cases as have proved fatal, the kidney was the only viscus diseased; the other two-thirds had disease of the heart and liver in addition to granulation of the kidneys. Of those cases of anasarca without coagulable urine which proved fatal, the heart was invariably greatly enlarged; and these were often complicated with ascites from disease of the liver." (P. 76.)

Dr. Seymour's treatment is similar to that usually recommended: he has found with Dr. Bright supertartrate of potash the best diuretic, and, like Dr. Osborne, the vapour bath the most useful diaphoretic.

The second part of this volume treats of Ascites. This may coexist with anasarca: when this latter arises from renal disease, in Dr. Seymour's experience ascites is uncommon; when the heart alone is diseased, "a collection of fluid occasionally occurs in the peritoneal cavity; but it is subordinate, from its smaller quantity, in importance to that contained in the cellular membrane." "The principal cause of ascites is disease of the liver." The most frequent appearances of the liver in these cases are, its diminution to one-half its natural size, its being very hard, and having its sharp edge blunt and rounded. The peritoneal coat is most frequently thickened either partially or occasionally entirely; the "partial thickening gives to the viscus the appearance of being puckered, not very unlike the lobulated structure of the calf's kidney." "Occasionally the whole peritoneal coat is thickened, of a milky-white colour, and the viscus beneath contracted," forming a hard and almost globular mass. In another condition of the liver attended with ascites, the peritoneal coat is transparent, but the secreting structure is nearly obstructed by the deposition of a reddish-white substance, by some believed to be lymph, the effect of inflammation, by others cholesterine; but "in either case, on a section of the liver, the whole seems to be made up of rounded masses, which, in some instances, can be separated from the cellular membrane which connects them."

The explanation which Dr. Seymour gives of the dropsical effusion consequent on these changes, is the pressure upon and obstruction of the minute branches of the vena portæ throwing back the blood on the trunk of the vein. It may happen, as Dr. Seymour supposes, that in some of these cases of contracted liver the peritoneal coat is thickened; but we are inclined from our own observation to agree with Dr. Carswell, who attributes the diminution in the size of the organ in such cases to disease of Glisson's capsule, which, as Mr. Kiernan has traced it, forms a sheath to the vessels and invests each of the lobules. Dr. Carswell* supposes this investing membrane to be converted into a fibrous tissue having great contractile powers: hence the diminished size of the liver, the pressure on the veins opposing the return of the blood from the viscera, and the constant consequence, ascites. Dr. Seymour justly opposes the idea that the effusion in such cases is owing to inflammation of the peritoneum, and the injurious deduction that it is to be combated with antiphlogistics; as he shows that in one series of cases the peritoneum is transparent. The external appearance of such a patient is thus graphically described.

"The distended abdomen is tense in every part, the swollen veins give to the whole a bluish appearance, while the hands and arms are wasted, the features drawn in and haggard, presenting the appearance of what has been termed 'facies hippocratica;' the legs are also often wasted, and indeed always, early in the disease, but at length the pressure of the immense body of fluid in the pelvis prevents the return of blood in the iliac veins, and the lower extremities become anasarcous. The pulse is quick and feeble; the tongue red, and rough with prominent papillæ, and sometimes aphthous; the urine is very scanty, and deposits a pink sediment, like the finest rouge; the thirst is insupportable; and the alvine excretions scanty and ill-coloured, the bowels being sometimes difficult to move, at others affected with diarrhœa." (P. 88.)

In such a condition of disease, we can only palliate by mild purgatives, light tonics to support the strength, and diuretics, particularly nitre, which relieves the craving thirst more than any remedy: many extol taraxacum, but Dr. Seymour never saw any obvious effect from its use beyond slight diuresis, or (when in large quantities,) relaxation of the bowels. In recent cases advantage is sometimes derived from rubbing a drachm of the linim. hydrargyri into the abdomen night and morning, and purging with calomel and senna with crystals of tartar every other day. Tapping has been recommended early, on the ground of diuretics acting better when

* See British and Foreign Review, No. IV. p. 463.

the distention is removed, which is sometimes the case; but Dr. Seymour does not advise it until purging and mercurial frictions have been tried. With this contracted and indurated liver the serum is commonly limpid; when it has been deeply tinged with blood, Dr. Seymour has invariably found some malignant tumour within the abdominal cavity, but he does not wish to be understood to say that such a disease cannot exist without bloody serum. Occasionally the serum is small in quantity and of the colour of whey, with shreds of lymph floating in it; this occurs in strumous patients at an early age, and is evidently the product of chronic inflammation modified by the constitutional disease. In some cases the effusion suddenly subsides, preceded by uncontrollable vomiting; or if tapping has been performed and does not again collect, violent pains in the bowels come on, with diarrhœa unmitigated by remedies, and in both instances death takes place.

"Where such cases have occurred, I have anxiously looked, after death, for some cause to explain these remarkable symptoms. A very unusual appearance has presented itself: the intestines are of a deep leaden blue colour throughout their whole extent, and the omentum of the same colour; the peritoneal coat covering the intestines thickened and opaque. Sometimes shreds of lymph, of no very recent formation, adhere to the convolutions, but in general the whole peritoneal covering of the intestines is only thickened equally throughout, and of this blue colour." (P. 95.)

"In every case in which such symptoms have preceded the termination of the patient's life, the ascites has been occasioned by the peculiar, hardened, and contracted state of the liver, which I have attempted to describe." (P. 96.)

Ascites may also arise from enlargement and induration of the liver and spleen, from repeated attacks of ague. If the patient is not constantly exposed to the miasmata, he may be generally permanently cured by active saline purgatives and mercurial frictions. The hydriodate of potash, in two or three grain doses twice daily, will sometimes succeed when other remedies fail.

The last chapter is devoted to the consideration of Ascites from that tuberculated accretion of the peritoneum so ably described by Dr. Baron. Dr. Cholmeley, of Guy's Hospital, first directed Dr. Seymour's attention to the fact that in this form of disease there is often vomiting of a deep leek-green liquid, precisely the colour of the beautiful fluor spar found at Alston in Cumberland. Dr. S. has never known this diagnostic symptom fail, but he does not remember to have seen this symptom present when the disease was accompanied with ascites, the evacuation preventing, probably, the serous effusion. As the effusion is the effect of chronic inflammation in strumous habits, neither tapping nor mercury given internally are advisable: if there should be tenderness on pressure, a few leeches or one bleeding may be necessary; but Dr. S. has relied on the hydriodate of potash, in two-grain doses, given twice daily in a draught of equal parts of cinnamon water and common water with a little syrup; and conium at night, if there is sleeplessness. Constipation is best relieved by half an ounce or six drachms of phosphate of soda given daily in a pint of beef-tea. This can be borne by stomachs which from organic disease reject every thing.

The remaining half of the volume before us is occupied with a translation of a work by Dr. F. C. Geromini on the cause and cure of dropsy. The object of Dr. G. is to prove that inflammation is uniformly the cause of dropsy; but, although Dr. Seymour gives publicity in this country to this exclusive theory, he prefixes an announcement that he considers the Italian physician has failed in establishing his principal conclusions. The Italian treatise is evidently the production of an able and learned man; but of one who leans too much to the opinion that in all instances it is useless to seek for more than one cause to explain one effect; a proposition sufficiently philosophical in physics, but not applicable to the explanation of diseases affecting our compound and most complex organization. The staple of the essay is of a totally different sort to that which is estimated in this country. No new facts, no set of cases, are brought forward; but, instead, there is much ingenious reasoning on the various symptoms of the disease, and on the opinions of those who have written upon it. It belongs wholly to the rational and not to the empirical school. In this respect it is a fair specimen of many Italian medical books; and, in a country where there is such a prevailing tendency, it can create no surprise that the doctrines of Brown found a more congenial soil than they did in the land of their nativity; or that the theories of Darwin's Zoonomia should still be quoted with serious earnestness. We confess we are surprised that Dr. Seymour should have taken the trouble to translate this book; or, having translated it, that he should have thought it advisable to add it to his own, thereby doubling its size and price, and adding little to its value. Dr. Geromini's work was published twenty years since!

To conclude: although we have not read Dr. Seymour's work with unqualified admiration, yet we hesitate not to say that he has written a useful book, one that may be perused with benefit and with pleasure; bearing ample evidence that its author writes from his own observation at the bed-side and the dissecting room; a circumstance which imparts a freshness, even when there is no originality, and which mere compilations, however excellent, never can possess. The real excellencies of the book make us regret the more its defects. We are at a loss to reconcile the importance which is attached to diagnosis, and the minute attention given to details of pathological anatomy, with so obvious a neglect (implying contempt) of the greatest discovery of our age,—the physical examination of the chest. Dr. Seymour writes fluently and apparently with ease; his style is colloquial rather than classical; and he occasionally introduces huge parentheses, which are sufficiently long and digressive for notes. His treatment is always cautious and judicious; and his minute observations on remedies bespeak much attention to that important part of the studies of the medical practitioner.

